# 181. Demographic and Geographic Epidemiology of *Staphylococcus aureus* Bacteremia using Geographic Information Systems Mapping and Spatial Statistics

**DOI:** 10.1093/ofid/ofab466.383

**Published:** 2021-12-04

**Authors:** Julia Marshall, Vance G Fowler, Felicia Ruffin, Paul Lantos, Christopher Timmins

**Affiliations:** 1 Duke University, Princeton, New Jersey; 2 Duke University Medical Center, Durham, North Carolina

## Abstract

**Background:**

Risk factors for community-associated *Staphylococcus aureus* bacteremia (SAB) are incompletely understood. We used Geographic Information Systems (GIS) and spatial statistics to analyze demographic and geographic epidemiology of SAB in the community.

**Methods:**

We used the *S. aureus* Bacteremia Group Prospective Cohort Study (SABG-PCS) at Duke University Medical Center to obtain demographic and clinical data. We used the American Community Survey and U.S. Census to supply neighborhood variables. Secular trends in demographic and clinical characteristics of SAB patients prospectively enrolled between 1995 and 2015 (n = 2478) were determined using linear regressions. To characterize spatial patterns in Methicillin-resistant *S. aureus* (MRSA) bacteremia compared to Methicillin-susceptible *S. aureus* (MSSA) bacteremia, we used GIS mapping and selected a subgroup of patients (n = 667) living in and around Durham County, North Carolina. We then created generalized additive models (GAMs) using this subgroup to detect geographic heterogeneities in probabilities of MRSA infections compared to MSSA infections.

**Results:**

We found evidence of changing demographic and clinical characteristics of SAB patients over the 21-year period. The proportion of infections acquired in the community increased significantly (p < 0.0001). However, we did not detect spatial heterogeneities of MRSA infections in Durham County. Patient location of residence was not significantly associated with antimicrobial-resistant infections. Patient age and year of hospital admission were the only statistically significant covariates in our spatial models.

**Conclusion:**

We utilized a novel method to analyze SAB in the community using GIS and spatial statistics. Future research should prioritize community transmission of *S. aureus* to identify robust risk factors for infection.

**Disclosures:**

**Vance G. Fowler, Jr., MD, MHS**, **Achaogen** (Consultant)**Advanced Liquid Logics** (Grant/Research Support)**Affinergy** (Consultant, Grant/Research Support)**Affinium** (Consultant)**Akagera** (Consultant)**Allergan** (Grant/Research Support)**Amphliphi Biosciences** (Consultant)**Aridis** (Consultant)**Armata** (Consultant)**Basilea** (Consultant, Grant/Research Support)**Bayer** (Consultant)**C3J** (Consultant)**Cerexa** (Consultant, Other Financial or Material Support, Educational fees)**Contrafect** (Consultant, Grant/Research Support)**Debiopharm** (Consultant, Other Financial or Material Support, Educational fees)**Destiny** (Consultant)**Durata** (Consultant, Other Financial or Material Support, educational fees)**Genentech** (Consultant, Grant/Research Support)**Green Cross** (Other Financial or Material Support, Educational fees)**Integrated Biotherapeutics** (Consultant)**Janssen** (Consultant, Grant/Research Support)**Karius** (Grant/Research Support)**Locus** (Grant/Research Support)**Medical Biosurfaces** (Grant/Research Support)**Medicines Co.** (Consultant)**MedImmune** (Consultant, Grant/Research Support)**Merck** (Grant/Research Support)**NIH** (Grant/Research Support)**Novadigm** (Consultant)**Novartis** (Consultant, Grant/Research Support)**Pfizer** (Grant/Research Support)**Regeneron** (Consultant, Grant/Research Support)**sepsis diagnostics** (Other Financial or Material Support, Pending patent for host gene expression signature diagnostic for sepsis.)**Tetraphase** (Consultant)**Theravance** (Consultant, Grant/Research Support, Other Financial or Material Support, Educational fees)**Trius** (Consultant)**UpToDate** (Other Financial or Material Support, Royalties)**Valanbio** (Consultant, Other Financial or Material Support, Stock options)**xBiotech** (Consultant)

